# FIFTEEN-YEAR TRENDS IN THE PATTERNS OF MEAL TIMING AND EATING FREQUENCY AMONG US ADULTS

**DOI:** 10.1093/geroni/igac059.1078

**Published:** 2022-12-20

**Authors:** Samaneh Farsijani, Ziling Mao, Anne Newman

**Affiliations:** University of Pittsburgh, Pittsburgh, Pennsylvania, United States; University of Pittsburgh, Pittsburgh, Pennsylvania, United States; University of Pittsburgh, Pittsburgh, Pennsylvania, United States

## Abstract

Understanding patterns of eating behavior may provide insights into contributors to obesity and metabolic diseases. To characterize trends in meal timing and frequency in US population age >19 years, we performed a serial cross-sectional analysis of 8 National Health and Nutrition Examination Survey (NHANES) cycles (2003-2018) on 34,470 adults (52.5% women and 21% black). Time of food and beverage intake was extracted from two 24-hour food recalls. The following meal timing measures were defined: 1) The time of the last and first calorie intake either from food or drink, 2) Eating window: The time elapsed between the first and last food intake, 3) Calorie midpoint time: The time when half of the calories for the day were consumed, 4) Late night eating: Consumption of ≥33% of total daily energy between 5:00pm and midnight, and 5) Eating frequency: Frequency of food or beverage consumption >0 kcal which were >15 minutes apart. From 2003 to 2018, survey-weighted mean (±SE) of eating window decreased from 12.21±0.06 to 12.02±0.05 hours/d (Ptrend=0.002). Time of the last calorie intake significantly decreased from 20.31±0.04 to 20.09±0.03 hours/d, while the time of first calorie intake and eating frequency remained unchanged. More than two-third of participants consumed ≥33% of total daily energy after 5:00pm. Over the 15-year span, composition of diet has also changed, including a decline in total calorie intake and percentage of energy from carbohydrate intake, while percentage of energy from dietary fats increased, Ptrend< 0.05. Patterns of eating behavior have changed in American adults over time.

